# Increasing nurses’ occupational well-being: the role of career shocks, job crafting and supervisor autonomy support

**DOI:** 10.1186/s12912-024-01955-4

**Published:** 2024-04-28

**Authors:** Ying Zhang, Xing Bu, Na Zhang

**Affiliations:** 1 School of Economics and Management, China University of Geosciences Beijing, Beijing, 100083 P.R. China; 2 School of Economics and Management, University of Science and Technology Beijing, Beijing, China; 3 School of Economics and Management, Beijing Information Science & Technology University, Beijing, China

**Keywords:** Occupational well-being, Positive career shocks, Negative career shocks, Job crafting, Supervisor autonomy support

## Abstract

**Background:**

This study aims to explore the influence of career shocks on nurses’ occupational well-being through job crafting and the moderating role of supervisor autonomy support.

**Method:**

The present study used a cross-sectional design. And the study included 714 frontline nurses in China, and we used structural equation modelling (SEM) to test our hypotheses.

**Results:**

Job crafting mediated both the relationship between positive career shocks and occupational well-being and the relationship between negative career shocks and occupational well-being. Supervisor autonomy support moderated the indirect relationships.

**Conclusions:**

Positive and negative career shocks could increase and impair nurses’ occupational well-being through job crafting, respectively. We contribute to helping nurses make sense of career shocks and preparing for career shocks, and hospital administrators and nurses’ direct supervisors can help nurse better cope with career shocks in attending job crafting activities and providing more autonomy supports.

## Background

Over the past 40 years, occupational well-being has been gaining momentum as it is a central psychological feeling in the occupational state [1, 2]. More and more healthcare organizations are acutely aware of the importance of nurses’ occupational well-being because frontline nurses experience high levels of burnout, sickness-related absenteeism, and leaving the profession [3, 4]. Researchers suggested that higher level of occupational well-being would be conducive to the stability and the development of the medical team, and it also would be helpful to promote the harmony of the nurse-patient relationship [5]. Thus, it is essential to understand how to increase nurses’ occupational well-being, and as such attract as well as retain qualified nurses.

Researchers began to explore the working environment factors which may increase nurses’ well-being [4], while what current models lack is an account of the role of unplanned or unexpected external events and how they impact nurses’ well-being. As we known, nurses are not only exposed to a number of occupational risks, but also some unexpected shock events. For example, the COVID-19 pandemic is a major career shock event for most people worldwide [6]. This shock event especially has significant effects on health care and frontline workers such as nurses who work around the clock to provide relevant and dedicated patient care. Therefore, it is necessary to devote increased attention to the role of career shock events in shaping nurses’ careers and work behaviours, especially nurses’ occupational well-being as well as the underline mechanism.

### Career shocks and occupational well-being

A career shock is defined as “a disruptive and extraordinary event that is, at least to some degree, caused by factors outside the focal individual’s control and that triggers a deliberate thought process concerning one’s career” [7]. Researchers suggested that career shock can be either positively or negatively valenced, for example, receiving a pay raise or promotion sooner than expected can be classified as a positive career shock, whereas having a mentor or colleague that was important to you leave the organization can be classified as a negative career shock [8]. In previous studies, positive career shocks have generally been associated with positive outcomes; and negative career shocks with negative outcomes [9].

Occupational well-being is defined as a positive assessment of one’s work life in the context of occupational health psychology [1], which has both personal and organizational implications for nurses. Occupational well-being reflects employees’ sense of competency, recognition and development aspirations related to their careers [10]. We examine the model from a job demands-resources (JD-R) perspective [11] which has been widely applied to understanding the antecedents of individuals’ well-being [12]. According to the JD-R model, the presence of job characteristics can be grouped into job demands and job resources. Job resources such as job autonomy and social support can stimulate personal growth and development and driver work engagement [11]. Job demands such as work overload, emotional job demands are all aspects that require sustained physical and/or psychological effort or skills to cope with and thus cause job burnout and many other negative outcomes [11].

Based on the conceptualization of career shocks, researchers have suggested that positive career shocks could represent a job resource because they can stimulate individuals’ growth and development [13]. Thus, according to the JD-R model, we proposed that positive shocks are positively related to individuals’ work engagement, which is a key indicator of their occupational well-being [14]. On the other hand, negative career shocks are more likely to represent hindrance demand because they may lead individuals to feel that their images (i.e., values and goals) are incompatible with their circumstances and cause them to take efforts to cope with those shocks and in turn be associated with lower occupational well-being [13]. Empirical evidence also provides support for our assumptions. For example, Mansur and Felix found that positive shocks had a positive effect on thriving, while negative career shocks had an indirect effect on thriving through career adaptability [15]. Thriving was defined as an individual psychological state involving the experience of vitality and learning [16], which share some similar characteristics with well-being. We then propose the following hypotheses:

H1: Positive career shocks are positively associated with occupational well-being.

H2: Negative career shocks are negatively associated with occupational well-being.

## The mediation role of job crafting

Job crafting refers to an individual’s volitional actions to shape, mould, or redefine their job in an attempt to improve their work experience [17]. It is a proactive behaviour strategy to cope with the dynamic changes of the organization, and could be triggered by both individual and organizational factors [17]. We proposed that career shocks would trigger nurses’ job crafting. Positive career shocks such as succeeding in a new project could provide a form of recognition and positive feedback [13]. Nurses may feel that they obtain approval, and may encourage them to change the design of their job and the social environment at work, which are the two forms of job crafting [18]. Experiencing positive career shocks could promote nurses try to improve their skills and abilities to make them more professional and competent, and they are more willing to change the quality or amount of interactions with others in ways that change one’s job. In contrast, negative career shocks can be direct signals of work pressures and uncertain conditions; based on those negative signals, individuals may be more likely to maintain the current situation and refuse to take any change let along redesign their job. In addition, when experiencing negative shocks, individuals put more cognitive and emotional efforts and energy to cope with them because such undesirable affective events would impede vitality and produce negative affective reactions [15]. In turn, individuals have a reduced capacity to engage in job crafting activities.

We then proposed that job crafting is positively related to occupational well-being. Through the job crafting process, individuals can enhance the meaning they attain from their work and in turn optimize their well-being [19]. There is already abundant evidence to support this relationship. For example, Slemp and Vella-Brodrick found that job crafting could predict employee intrinsic need satisfaction and in turn predicted employee psychological well-being and subjective well-being [20]. We proposed that experiencing positive career shocks may promote individuals to engage in job crafting and be positively associated with occupational well-being, while experiencing negative career shocks is negatively associated with occupational well-being through job crafting. We then propose the following hypotheses:

H3: The positive indirect relationship between positive career shocks and occupational well-being is mediated by job crafting.

H4: The negative indirect relationship between negative career shocks and occupational well-being is mediated by job crafting.

### Perceived supervisor autonomy support as moderator

Seibert and colleagues suggested a high quality relationship with supervisors is a vital step to maintain resilience and adaptability in one’s career and then could be effective strategies to respond to the inevitable career shocks [21]. We thus proposed that perceived supervisor autonomy support could act as a key moderator to influence the indirect effect of career shocks on nurses’ occupational well-being through job crafting. Supervisor autonomy support refers to a supervisor’s tendency to acknowledge employees’ perspectives, offer choices within specific rules and limits, provide meaningful feedback, encourage initiation and provide a rationale when individuals undertake a particular task [22].

Based on the conception of career shocks, unexpected events could trigger a thought process if individuals adopt job crafting activities [7] and the level of job crafting depends on the level of perceived supervisor autonomy support. When nurses perceive that their supervisors provide a high level of support, they will magnify the positive signal of positive career shocks, and they are more likely to make changes in job design and work environment, which in turn improve their well-being. This means that they could have a greater chance of obtaining suitable training and development opportunities and seeking job challenges and fit, which would lead to a less negative interpretation of the current situation and future career prospects [21]. Hence, under conditions of higher perceived supervisor autonomy support, the negative effect of negative career shocks on well-being through job crafting might be buffered. In addition, according to the proposition of the JD-R model, job resources can buffer the negative impact of job demands on work engagement and other positive outcomes [11]. And based on JD-R model, perceived supervisor autonomy support can be seen as a job resource because it can function in helping individuals to stimulate growth and personal development. Thus, supervisor autonomy support could represent a job resource which could buffer the negative effects of negative career shocks, and also strength the positive effect of positive effects. We then propose the following hypothesis:

H5: Perceived supervisor autonomy support moderates the mediated effect of career shocks on occupational well-being via job crafting, such that it will (a) strengthen the mediated relationship between positive career shocks and occupational well-being (b) and weaken the mediated relationship between negative career shocks and occupational well-being.

## Methods

### Data collection and participants

The sample included 727 frontline nurses in China. We relied on our personal connections and networks to recruit participants from three hospitals in China. We contacted the hospital managers of nurses directly or indirectly to ask them to randomly deliver the surveys to their nurses. The nurses completed the scales used to measure their career shocks, job crafting, perceived supervisor autonomy support, and occupational well-being. Each participant received 10 yuan after successfully completing the surveys. There were 714 effective matching responses (response rate of 98.2%). Data collection lasted from October to November 2021.

In the sample, 98.6% of the respondents were male. In terms of age, 18.8% were between 20 and 30 years old, 46.6% were between 31 and 40 years old, and the remainder were over 41 years old. In terms of education, 91.6% had a bachelor’s degree or higher, and the remainder had a high school-level education. In terms of working years, 10.8% have been working for less than 5 years, 26.2% have been working for approximately 5 to 10 years, 24.1% have been working for approximately 10 to 15 years, and the remainder have been working for more than 15 years. In terms of their titles, 14.7% were junior nurses, 36.3% were senior nurses, 33.8% were nurses-in-charge, 14.0% were associate chiefs of nursing, and 1.3% were head nurses.

### Measures

Unless otherwise noted, the responses to all items were measured on 7-point Likert-type scales, ranging from strongly disagree (1) to strongly agree (7).

*Career shocks.* We used three items each for negative and positive career shocks, which was used by Mansur and Felix [15]. The six items were taken from Seibert et al. [23] and Holtom et al. [24]. We adapted it to the nurses’ context. Respondents rated the degree to which each event impacted their career on a 5-point scale ranging from 0 (had not experienced the event; and thus had no impact) to 4 (had a large impact). The sample item for positive shocks was “Received a pay raise, promotion or desirable increase in responsibility sooner than expected”. The sample item used to measure negative shocks was “Your hospital went through a significant negative event such as a reduction in workforce, or major ethical scandal”. Following Mansur and Felix [15], we averaged the impact of the positive and negative shocks only for those who had experienced at least one.

*Job crafting.* We measured job crafting with four items developed by Leana et al. [25]. On a 5-point scale ranging from 1 (almost never) to 5 (very often), nurses were asked to rate how often they engaged in each of the listed behaviours. Sample items included “Change the way you do your job to make it easier to yourself” and “Introduce new approaches to improve my work.” The Cronbach’s alpha for the scale was 0.90.

*Perceived supervisor autonomy support*. We used Moreau and Mageau’s nine-item scale to measure participants’ perceptions of supervisor autonomy support [26]. Sample items included “My supervisor gives me many opportunities to make decisions in my work”. The Cronbach’s alpha for the scale was 0.94.

*Occupational well-being.* We measured it with a subscale of Chinese Employee Well-being that was developed by Huang [27]. The subscale has three dimensions (job competency, job ambition and job recognition) with 10 items to reflect employees’ occupational well-being. We adapted it to measure nurses’ occupational well-being. Sample items included “Be able to handle any problems in the work”. The Cronbach’s alpha for the scale was 0.95.

*Covariates.* We controlled for some demographic variables that may influence the relationships between the predictor and outcome variables, including age, gender (1 = male, 0 = female), education (1 = high school, 2 = college, 3 = masters and doctoral degree), years of working, and work titles (1 = junior nurse, 2 = senior nurse, 3 = nurse-in-charge, 4 = associate chief of nursing, 5 = head nurse).

### Data analysis

Descriptive statistics, such as means, standard deviations and correlations among the main variables were analysed by SPSS version 25.0. To test the research hypotheses, we then utilized structural equation modelling (SEM) using MPlus above and beyond the variables defined in previous steps.

## Results

### Common method variance testing

Because the data in the present study are self-report and came from the single source, we tried to control the common method variance by running a Harman’s single-factor test. The result of the PCA output displayed 29 unique factors explaining 69.77% of the overall variance. And the initial unrotated factor only accounted for 35.81% of the data variance, which is below 40%. As a result, the common method variance in this research fell within an acceptable threshold [28].

### Measurement model

We first performed confirmatory factor analyses (CFA) to ensure that all of the variables in our study had satisfactory discriminant validity. The results shows that the fit of the five-factor measurement model in which positive career shocks, negative career shocks, job crafting, supervisor autonomy support, and occupational well-being were represented as separate constructs was best compared to other models: χ2/df = 2.80, comparison fit index (CFI) = 0.97, and root mean square error of approximation (RMSEA) = 0.05.

### Correlation analysis

The descriptive statistics and correlations among variables are presented in Table 1. The results showed that positive career shocks were positively related to occupational well-being (*r* = 0.13**, *p* < 0.001) and job crafting (*r* = 0.16**, *p* < 0.001). And negative career shocks were negatively related to occupational well-being (*r* = − 0.15**, *p* < 0.001) and job crafting (*r* = − 0.12**, *p* < 0.001). Thus, both Hypotheses 1 and 2 were supported.

**Table 1:** *Correlations between Variables*

Variables	M	SD	1	2	3	4	5
1. Positive career shocks	1.70	1.14	-				
2. Negative career shocks	1.36	1.24	0.57**	-			
3.Supervisor autonomy support	4.75	1.32	0.15**	− 0.04	(0.94)		
4. Job crafting	3.77	0.81	0.16**	− 0.12**	0.32**	(0.90)	
5.Occupational well-being	5.47	1.06	0.13**	− 0.15**	0.38*	0.50**	(0.95)

Note : *n* = 714,**p < 0.01, *p < 0.05

### Testing of hypotheses

**Fig. 1:**
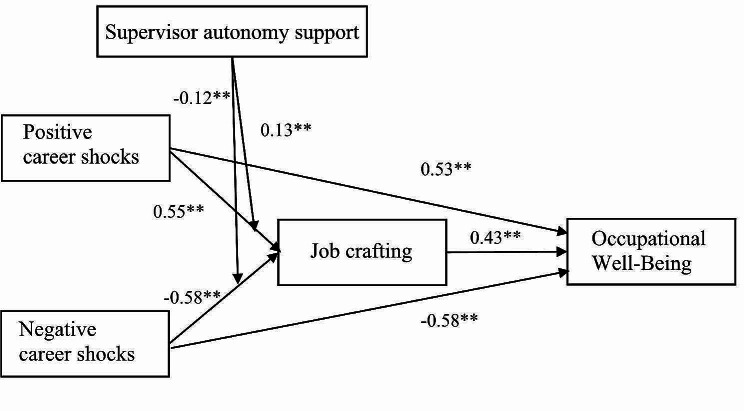
Summary of SEM results

We then used structural equation modelling (SEM) using MPlus to test our model. The results of the structural model are shown in Fig. 1; Table 2. As shown in Table 2, positive career shocks could positively predict occupational well-being (*b* = 0.53, SE = 0.08, *p* < 0.001). Similarly, negative career shocks could negatively predict occupational well-being (*b*= -0.58, SE = 0.07, *p* < 0.001). In addition, Table 2 shows that positive career shocks could positively predict job crafting (*b* = 0.55, SE = 0.12, *p* < 0.001), and negative career shocks could negatively predict job crafting (*b*= -0.58, SE = 0.12, *p* < 0.001).

**Table 2:** *Summary of Indirect Effects and Conditional Indirect Effects*

Path	Estimate	SE	*p*	95% credibility intervals
Positive career shocks →occupational well-being	0.53	0.08	< 0.001	[0.41, 0.65]
Negative career shocks →occupational well-being	-0.58	0.07	< 0.001	[-0.70, -0.46]
Positive career shocks →Job crafting	0.55	0.12	< 0.001	[0.35, 0.75]
Negative career shocks →Job crafting	-0.58	0.12	< 0.001	[-0.79, -0.41]
Positive career shocks →Job crafting→ occupational well-being	0.23	0.07	< 0.001	[0.15, 0.37]
Negative career shocks →Job crafting→ occupational well-being	-0.25	0.07	< 0.001	[-0.38, -0.16]
Positive career shocks ×supervisor autonomy support →Job crafting	0.13	0.04	< 0.001	[0.07, 0.19]
Negative career shocks ×supervisor autonomy support →Job crafting	-0.12	0.04	< 0.001	[-0.18, -0.05]
Positive career shocks ×supervisor autonomy support →Job crafting→ occupational well-being	0.04	0.01	< 0.001	[0.02, 0.06]
Negative career shocks ×supervisor autonomy support →Job crafting→ occupational well-being	-0.04	0.01	< 0.001	[-0.05, -0.02]

Bootstrapping procedures were used to test the significance of the mediating effect of job crafting between career shocks and occupational well-being. As seen in Table 2, job crafting mediated the relationship between positive career shocks and occupational well-being (coefficient = 0.23; 95% CI [0.15, 0.37]), as the credibility interval does not include zero. The results showed that the mediation effect of job crafting between negative career shocks and occupational well-being was also significant (coefficient = − 0.25; 95% CI [-0.38, − 0.16]) as the credibility interval did not include zero. Thus, both Hypotheses 3 and 4 were supported.

**Fig. 2:**
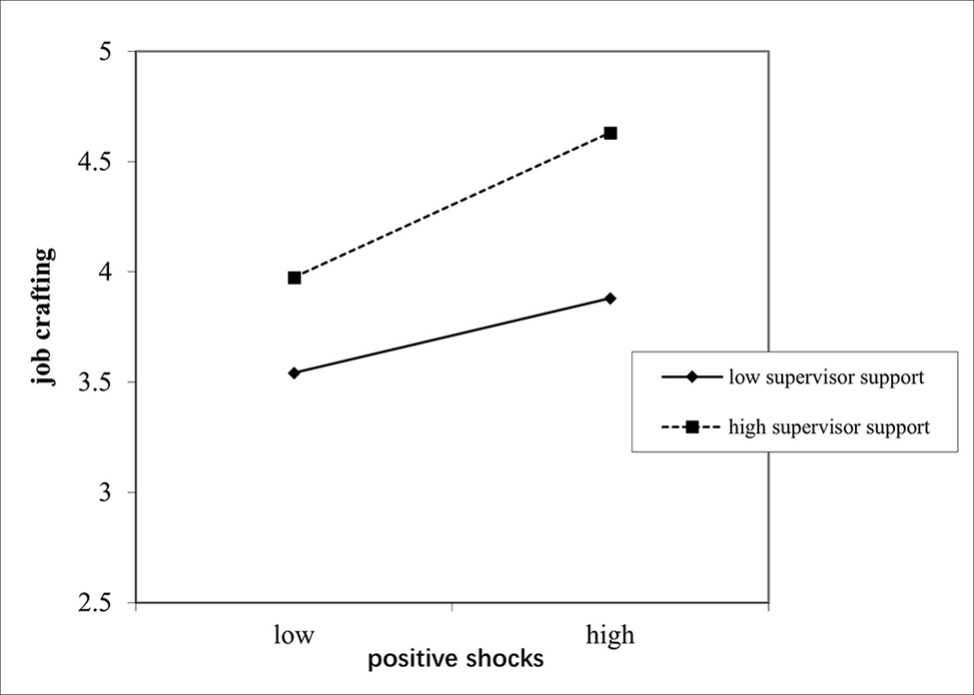
The moderating effects of supervisor autonomy support in relationship between positive career shocks and job crafting

**Fig. 3:**
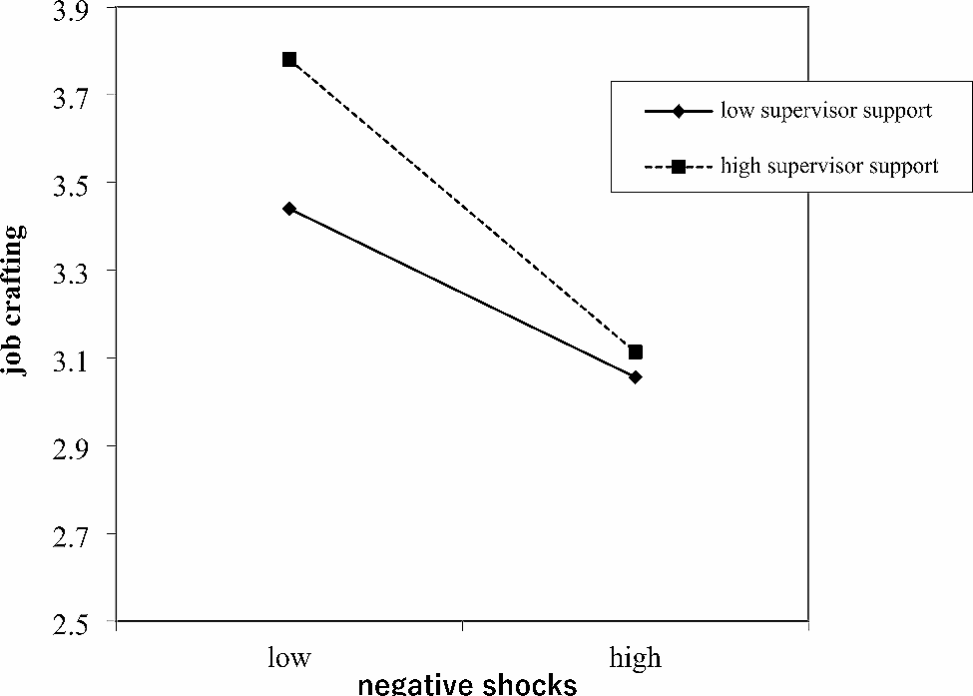
The moderating effects of supervisor autonomy support in relationship between negative career shocks and job crafting

We then tested Hypotheses 5a and 5b. The results from Table 2 showed that the overall path of the model was statistically significant (b = 0.04, SE = 0.01, *p* < 0.01). When perceived supervisor autonomy support was high, the indirect effect was significant (coefficient = 0.19; 95% CI [0.14, 0.23]). When perceived supervisor autonomy support was low, the indirect effect was also significant (coefficient = 0.07; 95% CI [0.03, 0.08]). To aid interpretation, we also plotted the interaction effects in Fig. 2. For Hypothesis 5b, the results from Table 2 showed that the overall path of the model was statistically significant (b= -0.04, SE = 0.01, *p* < 0.01). When perceived supervisor autonomy support was high, the indirect effect was significant (coefficient = − 0.17; 95% CI [-0.22, − 0.13]). When perceived supervisor autonomy support was low, the indirect effect was also significant (coefficient = − 0.08; 95% CI [-0.12, − 0.04]). To aid interpretation, we also plotted the interaction effects in Fig. 3.

## Discussion

The current study aimed to examine when and why career shocks can influence frontline nurses’ occupational well-being. And the overall findings of the present study contribute to the literature in several ways.

First, our study contributes to the career shock literature by linking it with nurses’ occupational well-being. Career theorists began to notice that planned or unplanned events outside employees’ immediate control often influence their career trajectory [23]. As a recent core debate on career sustainability, well-being is more likely to be influenced by career shocks. Career shocks represent a relevant antecedent of career development events. We found that shocks could not only impact individuals’ career trajectory but also trigger affects related to their occupation, such as the feeling of competency, ambition or recognition, which are all indices of occupational well-being. The results supported the hypothesis that positive shock events have a positive effect on the well-being of nurses and negative shocks have negative effects, which is consisted with the assumptions of career shocks [7]. It means that in nurses’ daily life, some unexpected events may influence their well-being, and the negative events would even reduce their occupational well-being.

Second, we further contribute to exploring the underlying mechanism of positive and negative career shock events on occupational well-being by considering the role of job crafting. This mediation results suggests that unplanned external career events can influence the intentional activity that people can adapt to improve their occupational well-being. Job crafting activity can allow nurses to redesign or change their work experience or environment to copy with or adapt to shocks and increase their enjoyment or satisfaction with their occupation. For example, succeeded in a new job or visible project may give nurses enough confidences and encourage nurses to change the design of their job and the social environment at work, which would further increase the enjoyment of their occupation. These results reinforce that career shock events not only have a nontrivial impact on one’s career paths of many people but also influence one’s work behaviours.

Finally, we included supervisor autonomy support as a job resource to examine the moderating effect. The result showed that perceived supervisor autonomy support could amplify the positive effect of positive career shocks and buffer the negative effect of negative career shocks. It means that when interfering or managing the influences of career shocks, supervisor autonomy support is of significant importance. SDT suggests that supervisor autonomy support is a situational factor assessed by employees’ perceptions of their managers that could enhance individuals’ basic psychological needs satisfaction and in turn increase their well-being [22]. Our research suggested that supervisor autonomy support could act as a job resource role to influence the relationship between career shocks and nurses’ occupational well-being, which also contributes to one of the propositions of the JD-R model [11].

### Practical implications

Our study can help to call for the nursing administration to pay more attention to the unexpected events that may happen in nurses’ daily life. And we also contribute to helping nurses make sense of career shocks and inform their career-related actions. Managers and career counselors could help nurses apply psychological strategies (i.e., managing distracting emotions) and behavioural strategies (i.e., undertaking suitable training and development opportunities) to encounter career shocks. More specifically, according to our results, hospital administrators can help nurse better cope with career shocks by trying to provide more autonomy support for their nurses, and nurses can actively attend more job crafting activities to decrease the negative effects of career shocks.

### Limitations

Although our research has several theoretical and practical contributions, it is not without limitations. First, the cross-sectional and single source data collection design of this study is insufficient to explore causal inferences. Future studies could advance to design a longitudinal or multisource investigation to examine the model of the present study. Second, previous studies on career shocks focus on exploring the influence of shock events on individuals’ career paths. Our results suggest that career shocks could also impact nurses’ well-being, but we did not explore whether shock events could also influence individuals’ other work behaviours, such as performance or creativity. Chen and colleagues [29] found that workplace event novelty and criticality interact to fuel employee creativity, and further research could explore the relationship between career shocks and creativity. Finally, the present study only considers supervisor autonomy support as a moderator, and further research could investigate other moderating variables (e.g., key resources, personnel characteristics), which would be helpful to help individuals copy career shocks.

## Conclusion

Findings from this study indicate that positive career shocks could increase nurses’ occupational well-being by promoting job crafting, while negative career shocks would impair nurses’ occupational well-being because they decrease job crafting. Thus, hospital administrators can use the findings to help nurses better cope with career shocks in attending job crafting activities, and a job crafting intervention could help nurses enhance the meaning they attain from their work and increase their well-being, which is important for nurses’ occupational health. Besides, we found that supervisor autonomy support is a pivotal factor in amplifying the positive effect and buffering the negative effect of career shocks; thus, the heads of nurses should try to provide autonomy support for their nurses, such as acknowledging nurses’ perspectives, encouraging their initiative, offering choices to them, and providing meaningful feedback.

## Data Availability

The datasets analysed during the current study are available from the corresponding author upon request.

## References

[1] Xu J, Xie B, Tang B (2020). Guanxi HRM practice and employees’ occupational well-being in China: a multi-level psychological process. Int J Environ Res Public Health.

[2] Meng Y, Luo X, Sun P, Luo Y, Wang Z, Wang L (2023). Occupational happiness of Civilian nurses in China: a cross-sectional study. BMC Nurs.

[3] Grabbe L, Higgins MK, Baird M, Craven PA, San Fratello S (2020). The Community Resiliency Model® to promote nurse well-being. Nurs Outlook.

[4] de Wijn AN, Fokkema M, van der Doef MP (2022). The prevalence of stress-related outcomes and occupational well-being among emergency nurses in the Netherlands and the role of job factors: a regression tree analysis. J Nurs Manag.

[5] Fang Z, Yang X (2018). Correlation between occupational well-being, job performance and doctor-patient relationship in medical worker. Chin J Health Psychol.

[6] Akkermans J, Richardson J, Kraimer M. The Covid-19 crisis as a career shock: implications for careers and vocational behavior. J Vocat Behav. 2020:103434. 10.1016/j.jvb.2020.103434 PMC7205633 32390655

[7] Akkermans J, Seibert SE, Mol ST (2018). Tales of the unexpected: integrating career shocks in the contemporary careers literature. SA J Ind Psychol.

[8] Akkermans J, Collings DG, Da Motta Veiga SP, Post C, Seibert S (2021). Toward a broader understanding of career shocks: exploring interdisciplinary connections with research on job search, human resource management, entrepreneurship, and diversity. J Vocat Behav.

[9] Hofer A, Spurk D, Hirschi A (2021). When and why do negative Organization-Related Career Shocks Impair Career Optimism? A conditional Indirect Effect Model. Career Dev Int.

[10] Zacher H, Rudolph CW (2021). Relationships between psychological contract breach and employee well-being and career‐related behavior: the role of occupational future time perspective. J Oogan Behav.

[11] Bakker AB, Demerouti E (2017). Job demands–resources theory: taking stock and looking forward. J Occup Health Psych.

[12] Demerouti E, Bakker AB (2023). Job demands-resources theory in times of crises: New propositions. Organ Psychol Rev.

[13] Kraimer ML, Greco L, Seibert SE, Sargent LD (2019). An investigation of academic career success: the new tempo of academic life. Acad Manag Learn Edu.

[14] Kilponen K, Huhtala M, Kinnunen U, Mauno S, Feldt T (2021). Illegitimate tasks in health care: illegitimate task types and associations with occupational well-being. J Clin Nurs.

[15] Mansur J, Felix B (2021). On lemons and lemonade: the effect of positive and negative career shocks on thriving. Career Dev Int.

[16] Spreitzer G, Sutcliffe K, Dutton J, Sonenshein S, Grant AM (2005). A socially embedded model of thriving at work. Organ Sci.

[17] Petrou P, Demerouti E, Schaufeli WB (2015). Job crafting in changing organizations: antecedents and implications for exhaustion and performance. J Occup Health Psych.

[18] Li J, Yang H, Weng Q, Zhu L (2023). How different forms of job crafting relate to job satisfaction: the role of person-job fit and age. Curr Psychol.

[19] Kilic E, Kitapci H (2023). Cognitive job crafting: an intervening mechanism between intrinsic motivation and affective well-being. Manag Res Rev.

[20] Slemp GR, Vella-Brodrick DA (2014). Optimising employee mental health: the relationship between intrinsic need satisfaction, job crafting, and employee well-being. J Happiness Stud.

[21] Seibert SE, Kraimer ML, Heslin PA (2016). Developing career resilience and adaptability. Organ Dyn.

[22] Deci EL, Olafsen AH, Ryan RM (2017). Self-determination theory in work organizations: the state of a science. Annu Rev Organ Psych.

[23] Seibert SE, Kraimer ML, Holtom BC, Pierotti AJ (2013). Even the best laid plans sometimes go askew: career self-management processes, career shocks, and the decision to pursue graduate education. J Appl Psychol.

[24] Holtom BC, Mitchell TR, Lee TW, Inderrieden EJ (2005). Shocks as causes of turnover: what they are and how organizations can manage them. Hum Resour Manage-US.

[25] Leana C, Appelbaum E, Shevchuk I (2009). Work process and quality of care in early childhood education: the role of job crafting. Acad Manage J.

[26] Moreau E, Mageau GA (2012). The importance of perceived autonomy support for the psychological health and work satisfaction of health professionals: not only supervisors count, colleagues too!. Motiv Emot.

[27] Huang L (2014). On the Dimensional structure of the employee Occupational Well-being in Chinese enterprises. J Cent Univ Finance Econ.

[28] Tehseen S, Ramayah T, Sajilan S (2017). Testing and controlling for common method variance: a review of available methods. J Manag Sci.

[29] Chen Y, Liu D, Tang G, Hogan TM. Workplace events and employee creativity: a multi๕tudy field investigation. Pers Psychol. 2020(2):1–26.

